# Supplementary material for: A Fraction of Escherichia coli Bacteria Induces an Increase in the Secretion of Extracellular Vesicle Polydispersity in Macrophages: Possible Involvement of Secreted EVs in the Diagnosis of COVID-19 with Bacterial Coinfections
Source: Int J Mol Sci. 2025 Apr 16;26(8):3741. doi: 10.3390/ijms26083741 (PMC12027499; doi:10.3390/ijms26083741)
Supplement: Supplementary file 1 [file ijms-26-03741-s001.zip › ijms-3390920-supplementary.pdf]

Supplementary Materials

Table S1. Protein identification in the *E. coli* fraction (SDS-SBMF) used to stimulated macrophage EVs secretion. Frag: SDS-PAGE Fragment number. U: Unused > 1.3 = 95% confidence. Significant peptide (95%). MALDI TOF TOF was used. Frag: SDS-PAGE Fragment used to the analysis.

Frag	U	Accession	Name	Peptides (95%)
F1	12.16	sp|P0CE48|EFTU2_ECOLI	Elongation factor Tu 2 OS=Escherichia coli (strain K12) GN=tufB PE=1 SV=1	8
	5.28	sp|P0ABB4|ATPB_ECOLI	ATP synthase subunit beta OS=Escherichia coli (strain K12) GN=atpD PE=1 SV=2	3
	4.74	sp|P02931|OMPF_ECOLI	Outer membrane protein F OS=Escherichia coli (strain K12) GN=ompF PE=1 SV=1	2
	4.08	sp|P31554|LPTD_ECOLI	LPS-assembly protein LptD OS=Escherichia coli (strain K12) GN=lptD PE=1 SV=2	2
	4	sp|P0AG67|RS1_ECOLI	30S ribosomal protein S1 OS=Escherichia coli (strain K12) GN=rpsA PE=1 SV=1	2
	2.28	sp|P0ABJ9|CYDA_ECOLI	Cytochrome bd-I ubiquinol oxidase subunit 1 OS=Escherichia coli (strain K12) GN=cydA PE=1 SV=1	1
F2	11.81	sp|P0CE48|EFTU2_ECOLI	Elongation factor Tu 2 OS=Escherichia coli (strain K12) GN=tufB PE=1 SV=1	9
	10	sp|P02931|OMPF_ECOLI	Outer membrane protein F OS=Escherichia coli (strain K12) GN=ompF PE=1 SV=1	6
	8.41	sp|P0AG67|RS1_ECOLI	30S ribosomal protein S1 OS=Escherichia coli (strain K12) GN=rpsA PE=1 SV=1	4
	8	sp|P0ABB4|ATPB_ECOLI	ATP synthase subunit beta OS=Escherichia coli (strain K12) GN=atpD PE=1 SV=2	4
	8	sp|P0A836|SUCC_ECOLI	Succinyl-CoA ligase [ADP-forming] subunit beta OS=Escherichia coli (strain K12) GN=sucC PE=1 SV=1	6
	6.52	sp|P0A940|BAMA_ECOLI	Outer membrane protein assembly factor BamA OS=Escherichia coli (strain K12) GN=bamA PE=1 SV=1	3
	6.15	sp|P0ABJ9|CYDA_ECOLI	Cytochrome bd-I ubiquinol oxidase subunit 1 OS=Escherichia coli (strain K12) GN=cydA PE=1 SV=1	3
	4.19	sp|P0AAI3|FTSH_ECOLI	ATP-dependent zinc metalloprotease FtsH OS=Escherichia coli (strain K12) GN=ftsH PE=1 SV=1	2
	4.06	sp|P33136|OPGG_ECOLI	Glucans biosynthesis protein G OS=Escherichia coli (strain K12) GN=mdoG PE=1 SV=1	2
	2.02	sp|P0AC41|SDHA_ECOLI	Succinate dehydrogenase flavoprotein subunit OS=Escherichia coli (strain K12) GN=sdhA PE=1 SV=1	2
	2	sp|P02919|PBPB_ECOLI	Penicillin-binding protein 1B OS=Escherichia coli (strain K12) GN=mrcB PE=1 SV=2	1
	2	sp|P33599|NUOCD_ECOLI	NADH-quinone oxidoreductase subunit C/D OS=Escherichia coli (strain K12) GN=nuoC PE=1 SV=3	1
	2	sp|P0ABB0|ATPA_ECOLI	ATP synthase subunit alpha OS=Escherichia coli (strain K12) GN=atpA PE=1 SV=1	1
	2	sp|P0A825|GLYA_ECOLI	Serine hydroxymethyltransferase OS=Escherichia coli (strain K12) GN=glyA PE=1 SV=1	1
	2	sp|P0A6F5|CH60_ECOLI	60 kDa chaperonin OS=Escherichia coli (strain K12) GN=groL PE=1 SV=2	2
	1.59	sp|P25526|GABD_ECOLI	Succinate-semialdehyde dehydrogenase [NADP(+)] GabD OS=Escherichia coli (strain K12) GN=gabD PE=1 SV=1	1
	1.35	sp|P0CB39|EPTC_ECOLI	Phosphoethanolamine transferase EptC	1

			OS=Escherichia coli (strain K12) GN=eptC PE=1 SV=1	
F3	36.07	sp|P0ABB4|ATPB_ECOLI	ATP synthase subunit beta OS=Escherichia coli (strain K12) GN=atpD PE=1 SV=2	24
	30	sp|P0ABB0|ATPA_ECOLI	ATP synthase subunit alpha OS=Escherichia coli (strain K12) GN=atpA PE=1 SV=1	19
	28.28	sp|P02930|TOLC_ECOLI	Outer membrane protein TolC OS=Escherichia coli (strain K12) GN=tolC PE=1 SV=3	22
	27.52	sp|P02943|LAMB_ECOLI	Maltoporin OS=Escherichia coli (strain K12) GN=lamB PE=1 SV=1	33
	12.05	sp|P0ABJ9|CYDA_ECOLI	Cytochrome bd-I ubiquinol oxidase subunit 1 OS=Escherichia coli (strain K12) GN=cydA PE=1 SV=1	7
	10.03	sp|P0CE48|EFTU2_ECOLI	Elongation factor Tu 2 OS=Escherichia coli (strain K12) GN=tufB PE=1 SV=1	10
	0	sp|P0CE47|EFTU1_ECOLI	Elongation factor Tu 1 OS=Escherichia coli (strain K12) GN=tufA PE=1 SV=1	10
	6	sp|P02931|OMPF_ECOLI	Outer membrane protein F OS=Escherichia coli (strain K12) GN=ompF PE=1 SV=1	3
	4	sp|P0ABC7|HFLK_ECOLI	Modulator of FtsH protease HflK OS=Escherichia coli (strain K12) GN=hflK PE=1 SV=1	2
	2.01	sp|P68187|MALK_ECOLI	Maltose/maltodextrin import ATP-binding protein MalK OS=Escherichia coli (strain K12) GN=malK PE=1 SV=1	1
	2	sp|P28903|NRDD_ECOLI	Anaerobic ribonucleoside-triphosphate reductase OS=Escherichia coli (strain K12) GN=nrdD PE=1 SV=2	1
	2	sp|Q46814|XDHD_ECOLI	Probable hypoxanthine oxidase XdhD OS=Escherichia coli (strain K12) GN=xdhD PE=3 SV=1	1
	2	sp|P00393|DHNA_ECOLI	NADH dehydrogenase OS=Escherichia coli (strain K12) GN=ndh PE=1 SV=2	1
	2	sp|P60872|YIDE_ECOLI	Putative transport protein YidE OS=Escherichia coli (strain K12) GN=yidE PE=3 SV=1	1
	1.51	sp|P0AA78|EXUT_ECOLI	Hexuronate transporter OS=Escherichia coli (strain K12) GN=exuT PE=1 SV=2	1
F4	26.72	sp|P02931|OMPF_ECOLI	Outer membrane protein F OS=Escherichia coli (strain K12) GN=ompF PE=1 SV=1	40
F5	20	sp|P0A910|OMPA_ECOLI	Outer membrane protein A OS=Escherichia coli (strain K12) GN=ompA PE=1 SV=1	13
	8	sp|P02931|OMPF_ECOLI	Outer membrane protein F OS=Escherichia coli (strain K12) GN=ompF PE=1 SV=1	4
F6	12.13	sp|P02931|OMPF_ECOLI	Outer membrane protein F OS=Escherichia coli (strain K12) GN=ompF PE=1 SV=1	8
	10	sp|P0A910|OMPA_ECOLI	Outer membrane protein A OS=Escherichia coli (strain K12) GN=ompA PE=1 SV=1	5
	4.77	sp|P0A927|TSX_ECOLI	Nucleoside-specific channel-forming protein tsx OS=Escherichia coli (strain K12) GN=tsx PE=1 SV=1	3
	2	sp|P0A7L0|RL1_ECOLI	50S ribosomal protein L1 OS=Escherichia coli (strain K12) GN=rplA PE=1 SV=2	1
	2	sp|P0ABJ9|CYDA_ECOLI	Cytochrome bd-I ubiquinol oxidase subunit 1 OS=Escherichia coli (strain K12) GN=cydA PE=1 SV=1	1
F7	6.13	sp|P02931|OMPF_ECOLI	Outer membrane protein F OS=Escherichia coli (strain K12) GN=ompF PE=1 SV=1	3
	3.85	sp|P0AG55|RL6_ECOLI	50S ribosomal protein L6 OS=Escherichia coli (strain K12) GN=rplF PE=1 SV=2	2
	2.77	sp|P62399|RL5_ECOLI	50S ribosomal protein L5 OS=Escherichia coli	1

			(strain K12) GN=rplE PE=1 SV=2	
	1.5	sp|P0ABJ9|CYDA_ECOLI	Cytochrome bd-I ubiquinol oxidase subunit 1 OS=Escherichia coli (strain K12) GN=cydA PE=1 SV=1	1
F8	16.04	sp|P0A917|OMPX_ECOLI	Outer membrane protein X OS=Escherichia coli (strain K12) GN=ompX PE=1 SV=1	8
	4	sp|P69776|LPP_ECOLI	Major outer membrane lipoprotein Lpp OS=Escherichia coli (strain K12) GN=lpp PE=1 SV=1	2
	2.01	sp|P69411|RCSF_ECOLI	Outer membrane lipoprotein RcsF OS=Escherichia coli (strain K12) GN=rcsF PE=1 SV=1	1
	2	sp|P0ADY7|RL16_ECOLI	50S ribosomal protein L16 OS=Escherichia coli (strain K12) GN=rplP PE=1 SV=1	1
	2	sp|P0AEU7|SKP_ECOLI	Chaperone protein Skp OS=Escherichia coli (strain K12) GN=skp PE=1 SV=1	2
	2	sp|P0A7J3|RL10_ECOLI	50S ribosomal protein L10 OS=Escherichia coli (strain K12) GN=rplJ PE=1 SV=2	1
F9	12	sp|P0ADW3|YHCB_ECOLI	Inner membrane protein YhcB OS=Escherichia coli (strain K12) GN=yhcB PE=1 SV=2	7
	12	sp|P02931|OMPF_ECOLI	Outer membrane protein F OS=Escherichia coli (strain K12) GN=ompF PE=1 SV=1	9
	10	sp|P68919|RL25_ECOLI	50S ribosomal protein L25 OS=Escherichia coli (strain K12) GN=rplY PE=1 SV=1	5
	8.01	sp|P0A905|SLYB_ECOLI	Outer membrane lipoprotein SlyB OS=Escherichia coli (strain K12) GN=slyB PE=2 SV=1	9
	8	sp|P61175|RL22_ECOLI	50S ribosomal protein L22 OS=Escherichia coli (strain K12) GN=rplV PE=1 SV=1	6
	8	sp|P0A7K6|RL19_ECOLI	50S ribosomal protein L19 OS=Escherichia coli (strain K12) GN=rplS PE=1 SV=2	4
	6.06	sp|P0A7R5|RS10_ECOLI	30S ribosomal protein S10 OS=Escherichia coli (strain K12) GN=rpsJ PE=1 SV=1	3
	6.01	sp|P0A910|OMPA_ECOLI	Outer membrane protein A OS=Escherichia coli (strain K12) GN=ompA PE=1 SV=1	3
	6.01	sp|P0A7S9|RS13_ECOLI	30S ribosomal protein S13 OS=Escherichia coli (strain K12) GN=rpsM PE=1 SV=2	3
	4	sp|P0A7R9|RS11_ECOLI	30S ribosomal protein S11 OS=Escherichia coli (strain K12) GN=rpsK PE=1 SV=2	2
	4	sp|P0AG48|RL21_ECOLI	50S ribosomal protein L21 OS=Escherichia coli (strain K12) GN=rplU PE=1 SV=1	3
	4	sp|P0A7J7|RL11_ECOLI	50S ribosomal protein L11 OS=Escherichia coli (strain K12) GN=rplK PE=1 SV=2	2
	4	sp|P0A7T7|RS18_ECOLI	30S ribosomal protein S18 OS=Escherichia coli (strain K12) GN=rpsR PE=1 SV=2	2
	4	sp|P0C054|IBPA_ECOLI	Small heat shock protein IbpA OS=Escherichia coli (strain K12) GN=ibpA PE=1 SV=1	2
	4	sp|P0AG44|RL17_ECOLI	50S ribosomal protein L17 OS=Escherichia coli (strain K12) GN=rplQ PE=1 SV=1	2
	4	sp|P0A7U3|RS19_ECOLI	30S ribosomal protein S19 OS=Escherichia coli (strain K12) GN=rpsS PE=1 SV=2	2
	4	sp|P0ADZ7|YAJC_ECOLI	UPF0092 membrane protein YajC OS=Escherichia coli (strain K12) GN=yajC PE=1 SV=1	3
	4	sp|P0A917|OMPX_ECOLI	Outer membrane protein X OS=Escherichia coli (strain K12) GN=ompX PE=1 SV=1	2
	4	sp|P69776|LPP_ECOLI	Major outer membrane lipoprotein Lpp OS=Escherichia coli (strain K12) GN=lpp PE=1 SV=1	5
	4	sp|P60624|RL24_ECOLI	50S ribosomal protein L24 OS=Escherichia coli (strain K12) GN=rplX PE=1 SV=2	2
	2.16	sp|P68679|RS21_ECOLI	30S ribosomal protein S21 OS=Escherichia coli (strain K12) GN=rpsU PE=1 SV=2	1
	2.05	sp|P0ADY3|RL14_ECOLI	50S ribosomal protein L14 OS=Escherichia coli (strain K12) GN=rplN PE=1 SV=1	1
	2.04	sp|P0A7U7|RS20_ECOLI	30S ribosomal protein S20 OS=Escherichia coli (strain K12) GN=rpsT PE=1 SV=2	1

	2.03	sp|P0A7R1|RL9_ECOLI	50S ribosomal protein L9 OS=Escherichia coli (strain K12) GN=rplI PE=1 SV=1	1
	2.01	sp|P0A7L8|RL27_ECOLI	50S ribosomal protein L27 OS=Escherichia coli (strain K12) GN=rpmA PE=1 SV=2	1
	2	sp|P0C018|RL18_ECOLI	50S ribosomal protein L18 OS=Escherichia coli (strain K12) GN=rplR PE=1 SV=1	1
	2	sp|P06715|GSHR_ECOLI	Glutathione reductase OS=Escherichia coli (strain K12) GN=gor PE=1 SV=1	1
	1.34	sp|P0ACF0|DBHA_ECOLI	DNA-binding protein HU-alpha OS=Escherichia coli (strain K12) GN=hupA PE=1 SV=1	1

Table S2. Secretion of EVs by macrophages analyzes by Fiji and the EVAnalyzer plujing (version 8.1.3 beta). Identical thresholds 10, Thershold method 'Li', Min circularity: 0.5, Filter Type EV-GFP and identical settings for all conditions that are compared were used on confocal images with FITC fluorescence channel. AU: Arbitrary units. Valid: number of EVs detected. The table is representative of the amount of EVs near and/or anchored per each macrophage cytoplasmic membrane surface. In unstimulated macrophages, the cells that secrete EVs were sought.

Stimulated EV_FITC (EV_GFP)				
**area size (AU)**	**intensity**	**circularity [0-1]**	**valid**	**Invalid**
17.55	11.35	0.95	11.00	0.00
26.64	12.05	0.87	22.00	0.00
27.78	11.35	0.89	18.00	0.00
**Unstimulated macrophages that were spontaneously secreting VEs EV_FITC (EV_GFP)**				
**area size (AU)**	**intensity**	**circularity [0-1]**	**valid**	**Invalid**
3.50	11.83	0.97	4.00	0.00
5.09	11.53	0.95	11.00	0.00
4.	12.70	0.98	6.00	0.00

Table S3. Protein identification in the mix of EVs (short and large) released by stimulated macrophage. U: Unused > 1.3 = 95 % confidence. Significant peptide (95%). MALDI TOF TOF was used. Frag: SDS-PAGE Fragment used to the analysis.

Frag	U	Accession	Name	Peptides (95%)
F1	4	sp|P07724|ALBU_MOUSE	Serum albumin OS=Mus musculus GN=Alb PE=1 SV=3	2
	2	tr|E9Q5F4|E9Q5F4_MOUSE	Actin, cytoplasmic 1 (Fragment) OS=Mus musculus GN=Actb PE=1 SV=1	1
	2	sp|P28828|PTPRM_MOUSE	Receptor-type tyrosine-protein phosphatase mu OS=Mus musculus GN=Ptprm PE=2 SV=2	1
	2	sp|Q9CQ21|MCTS2_MOUSE	Malignant T-cell-amplified sequence 2 OS=Mus musculus GN=Mcts2 PE=2 SV=1	1
	2	sp|Q99NB8|UBQL4_MOUSE	Ubiquilin-4 OS=Mus musculus GN=Ubqln4 PE=1 SV=1	1
F2	10.89	sp|P63017|HSP7C_MOUSE	Heat shock cognate 71 kDa protein OS=Mus musculus GN=Hspa8 PE=1 SV=1	5
	2.66	sp|P26041|MOES_MOUSE	Moesin OS=Mus musculus GN=Msn PE=1 SV=3	1
	2.02	sp|P07724|ALBU_MOUSE	Serum albumin OS=Mus musculus GN=Alb PE=1 SV=3	1
	2	sp|P52480|KPYM_MOUSE	Pyruvate kinase PKM OS=Mus musculus GN=Pkm PE=1 SV=4	1
F3	2.45	sp|P63260|ACTG_MOUSE	Actin, cytoplasmic 2 OS=Mus musculus GN=Actg1 PE=1 SV=1	5
	1.96	tr|Q99LB4|Q99LB4_MOUSE	Capping protein (Actin filament), gelsolin-like OS=Mus musculus GN=Capg PE=1 SV=1	1
F4	12	sp|P17751|TPIS_MOUSE	Triosephosphate isomerase OS=Mus musculus GN=Tpi1 PE=1 SV=4	6

	10.66	sp|Q00623|APOA1_MOUSE	Apolipoprotein A-I OS=Mus musculus GN=Apoa1 PE=1 SV=2	6
	6.03	sp|Q99PT1|GDIR1_MOUSE	Rho GDP-dissociation inhibitor 1 OS=Mus musculus GN=Arhgdia PE=1 SV=3	4
	6.02	tr|A0A0A0MQF6|A0A0A0MQF6_MOUSE	Glyceraldehyde-3-phosphate dehydrogenase OS=Mus musculus GN=Gapdh PE=1 SV=1	3
	6	tr|Q8C253|Q8C253_MOUSE	Galectin OS=Mus musculus GN=Lgals3 PE=1 SV=1	3
	2.02	sp|P48036|ANXA5_MOUSE	Annexin A5 OS=Mus musculus GN=Anxa5 PE=1 SV=1	1
	2.02	sp|P02089|HBB2_MOUSE	Hemoglobin subunit beta-2 OS=Mus musculus GN=Hbb-b2 PE=1 SV=2	2
	2	sp|P62242|RS8_MOUSE	40S ribosomal protein S8 OS=Mus musculus GN=Rps8 PE=1 SV=2	1
	2	sp|Q8BFU2|H2A3_MOUSE	Histone H2A type 3 OS=Mus musculus GN=Hist3h2a PE=1 SV=3	1
	2	sp|Q9JLZ6|HIC2_MOUSE	Hypermethylated in cancer 2 protein OS=Mus musculus GN=Hic2 PE=2 SV=4	1
	2	sp|P45591|COF2_MOUSE	Cofilin-2 OS=Mus musculus GN=Cfl2 PE=1 SV=1	1
	2	tr|J3QK04|J3QK04_MOUSE	MCG67952 OS=Mus musculus GN=Gm7808 PE=4 SV=1	1
	2	tr|E9Q5F4|E9Q5F4_MOUSE	Actin, cytoplasmic 1 (Fragment) OS=Mus musculus GN=Actb PE=1 SV=1	1
	2	sp|Q9WUU7|CATZ_MOUSE	Cathepsin Z OS=Mus musculus GN=Ctsz PE=2 SV=1	1
	2	sp|P62908|RS3_MOUSE	40S ribosomal protein S3 OS=Mus musculus GN=Rps3 PE=1 SV=1	1
	2	tr|Q91VB8|Q91VB8_MOUSE	Alpha globin 1 OS=Mus musculus GN=haemaglobin alpha 2 PE=1 SV=1	2
	2	sp|O88569|ROA2_MOUSE	Heterogeneous nuclear ribonucleoproteins A2/B1 OS=Mus musculus GN=Hnrnpa2b1 PE=1 SV=2	1
F7	18.06	sp|P07724|ALBU_MOUSE	Serum albumin OS=Mus musculus GN=Alb PE=1 SV=3	13
	2	sp|P63260|ACTG_MOUSE	Actin, cytoplasmic 2 OS=Mus musculus GN=Actg1 PE=1 SV=1	1
	2	sp|P32261|ANT3_MOUSE sp|P32261|ANT3_MOUSE	Antithrombin-III OS=Mus musculus GN=Serpinc1 PE=1 SV=1	1

Table S4. Bacterial co-infections analyzed in COVID-19 patients. Infection with the following bacterial species was evaluated: *Streptococcus pneumoniae, Staphylococcus aureus, Streptococcus aureus, Klebsiella pneumoniae, Pseudomonas aeruginosa, Acinetobacter baumannii.*

BACTERIAS	SARS-CoV-2 +		CLINICAL DATA		
	N	%	OXYGEN SATURATION (%)	TEMPERATURE (°C)	TAC (CORADS)
None	1	1.2	89	37	5
Staphylococcus aureus	2	2.4	70	38	5
Streptococcus pneumoniae	5	6	*68*	38.1	5
Acinetobacter baumannii and Streptococcus pneumoniae	1	1.2	90	*36*	*5*
Klebsiella pneumoniae and Staphylococcus aureus	2	2.4	75.5	37.2	5
Klebsiella pneumoniae and Streptococcus pneumoniae	2	2.4	76	39.1	5
Staphylococcus aureus, Streptococcus pneumoniae	38	45.8	67	37.5	5
Acinetobacter baumannii, Staphylococcus aureus and Streptococcus pneumoniae.	2	2.4	75	36.6	5
Staphylococcus aureus, Streptococcus pneumoniae and Pseudomonas aeruginosa.	2	1.2	83	37.3	5

Staphylococcus aureus, Streptococcus pneumoniae and Klebsiella pneumoniae.	23	28.9	67	37.5	4.5
Staphylococcus aureus, Streptococcus pneumoniae, Klebsiella pneumoniae and Acinetobacter baumannii.	2	2.4	63	38.1	5
Staphylococcus aureus, Streptococcus pneumoniae, Klebsiella pneumoniae and Pseudomonas aeruginosa.	3	3.6	84.3	36.4	5

Table S5. Analysis of bacterial coinfection in COVID-19 patients via PCR. Detection of diverse bacterias was evaluated using probes and primers by: *Streptococcus pneumoniae, Staphylococcus aureus, Streptococcus aureus, Klebsiella pneumoniae, Pseudomonas aeruginosa, Acinetobacter baumannii*.

Specie	Forward	Reverse	Probe
Staphylococcus aureus	GGGTGAGTAACACGTGGATAAC	CAGCGCGGATCCATCTATAAG	ACTGGGATAACTTCGGGAAACCGG
Streptococcus pneumoniae	CGTCAGTTACAAGCCAGAGAG	GGCTGTGGCTTAACCATAGTAG	ACGCATTTCACCGCTACACATGGA
Acinetobacter baumannii	GGGCAGGTACATTGGCTTTA	ATCTGCACCAGAAGGACCAC	TGGCAAGTGTTTGCCATTG
Klepsiella pneumoniae,	CAGAAGAAGCACCGGCTAAC	CTACGCATTTCACCGCTACA	ACGGAGGGTGCAAGCGTTAA
Pseudomonas aeruginosa,	CAACTGCCTGGTCATCTTCA	TGTTCTTCACCACCTTGACG	GCATGAAGATCGGCGTCATG
